# Impact of Intra-Abdominal Adhesion on Dialysis Outcome in Peritoneal Dialysis Patients

**DOI:** 10.1155/2018/1978765

**Published:** 2018-09-25

**Authors:** Ben-Chung Cheng, Nai-Wen Tsai, Yun-Ru Lai, Chin-Cheng Huang, Cheng-Hsien Lu

**Affiliations:** ^1^Departments of Biological Science, National Sun Yat-Sen University, Kaohsiung, Taiwan; ^2^Department of Nephrology, Chang Gung Memorial Hospital-Kaohsiung Medical Center, Chang Gung University College of Medicine, Kaohsiung, Taiwan; ^3^Department of Neurology, Chang Gung Memorial Hospital-Kaohsiung Medical Center, Chang Gung University College of Medicine, Kaohsiung, Taiwan; ^4^Departments of Neurology, Xiamen Chang Gung Memorial Hospital, Xiamen, Fujian, China

## Abstract

**Background:**

Peritoneal dialysis (PD) is an increasingly popular therapeutic option for patients with advanced renal failure. However, intra-abdominal adhesions (IAA) represent a major unsolved problem in adequate PD performance. In this study, we investigated the role of previous abdominal surgery on the presence of subsequent IAA as well as outcomes in those patients with PD who had subsequent IAA.

**Methods:**

Two hundred and two patients who received continuous ambulatory peritoneal dialysis were prospectively enrolled in this study. We compared the PD adequacy indices and outcomes for technical failure in patients with and without subsequent IAA at presentation and a minimum of 2 years of follow-up.

**Results:**

Subsequent IAA accounted for 19% (38/202) of patients. Patients who had previous abdominal surgery had higher risks of subsequent IAA especially those patients who had higher mean ages (P=0.023). PD adequacy indices including both 24-hour dialysate volume and peritoneal WCcr L/week/1.73 m^2^ were significantly lower in patients who had, as compared to those who did not have subsequent IAA (P=0.003 and 0.018, respectively). Although patients who had subsequent IAA had decreased PD adequacy, the development of technical failures during PD maintenance did not show significant differences at the 2-year minimum follow-up study.

**Conclusions:**

Subsequent IAA is not rare, especially in high-risk patients including those with previous abdominal surgery and higher mean ages. Although decreased PD adequacy after IAA was found, the development of technical failures was not significantly different at the 2-year minimum follow-up study.

## 1. Introduction

Peritoneal dialysis (PD) is an effective home modality for alternative renal replacement therapy in the management of patients with end stage renal disease (ESRD) [1, 2]. There are several clear advantages offered by PD therapy in terms of preservation of residual renal function, patient satisfaction, and the promotion of an optimal quality of life [3–8]. Although variable factors influence the successful maintenance of PD therapy including a well-functioning peritoneal catheter, adequate peritoneal membrane properties, supportive familial system, and good patient adherence to therapy, nephrologists are often plagued by the presence of a history of abdominal surgery. However, patients performing PD are subject to numerous noninfectious complications such as obstruction-related catheter malfunctions [9, 10]. Furthermore, catheter obstructions may be due to any of a variety of causes including intraluminal obstruction by a fibrin or clot, as well as distended loops of bowel and adhesions due to constipation and prior peritonitis or surgery, respectively. Peritoneal adhesions occur in 70-90% of abdominal operations [11, 12], with a higher incidence than the nondialysis population [13–17]. It may cause the catheter to be trapped in a loculated compartment, and surgical lysis or catheter repositioning may be indicated for adequate PD performance.

To our knowledge, a limited number of studies have examined the relationships between clinical outcome, complications, and preservation of residual renal function among PD patients, regardless of the presence of subsequent intra-abdominal adhesions (IAA) [18, 19]. This hospital-based study may provide accurate information on the relative frequency of subsequent IAA after catheter insertion and their effects on dialysis adequacy and mortality. There is also a need for better delineation of the role of previous abdominal surgery on the presence of subsequent IAA and outcomes in this those patients who had subsequent IAA after catheter insertion. Therefore, the purpose of our study was to investigate the relationship between prior abdominal surgical procedures and subsequent IAA and determine the effects of subsequent IAA on both dialysis adequacy and outcome in PD patients. This report aimed to demonstrate the consequences of intra-abdominal adhesion in patients with PD therapy with respect to long-term follow-up of PD adequacy indices and clinical outcomes.

## 2. Materials and Methods

### 2.1. Patients and Hospital Setting

All patients with maintenance PD were consecutively recruited from the nephrology outpatient department of the Kaohsiung Chang Gung Memorial Hospital (KCGMH) in Southern Taiwan. All data were captured retrospectively from nursing and clinical records at the KCGMH PD unit. Two hundred two consecutive procedures for catheter implantation of PD cases were retrospectively enrolled into the present study. Experienced surgeons in our institution performed all the PD catheter insertions. Catheter implantations were performed using laparoscopic or conventional approaches, as previously described [20–22]* and the IAA was confirmed by laparoscopy.* The hospital's Institutional Review Committees on Human Research approved the study protocol (CGMH 100-2661B).

### 2.2. Clinical Assessment

PD adequacy indices, including Kt/V urea, weekly creatinine clearance (WCcr), measures of nutritional status (albumin, body mass index [BMI]), and normalized protein catabolic rate (nPCR) were measured at one month, for a total of three consecutive evaluations after PD initiation during the 2-year minimum follow up study between patients with or without subsequent IAA. Postoperative complication rates were compared between patients with and without a history of abdominal surgery. We defined catheter survival time as the duration of continuous catheter use and catheter failure was defined as the removal of the catheter for mechanical and infectious complications such as pericannular leak, flow obstruction, and severe peritonitis. Although catheter loss due to death or transplantation was censored, it was counted as survived because these catheters did not have inherent problems and were in working condition when removed. Infectious complications included peritonitis, exit site or tunnel infections, and admission events during follow up were summarized during the study period. Evaluation of dialysis adequacy and outcomes was performed during a 2-year minimum follow-up period, which may be terminated by death.

### 2.3. Statistical Analysis

Two separate statistical analyses were performed. Categorical variables were compared using the Chi-square or Fisher's exact tests. Continuous variables within the two groups were compared using the independent t-test for parametric data and Mann-Whitney U test for nonparametric data. First, repeated measures of ANOVA were used to compare patients with and without subsequent intra-abdominal adhesions at two different time points (at presentation after catheter insertion and after a minimum of 2 years of follow-up). Analysis of covariance (ANCOVA) was used to compare the groups (PD patients with or without subsequent intra-abdominal adhesions) after controlling for potential confounding variables. Levene's test of equality of error variance was used to ensure that equal variance existed in both groups. For comparison of the PD adequacy indices between the groups, we employed ANCOVA with or without prior abdominal surgery, as potential confounding variables. Second, the survival curves for technical failure between the two patient groups with or without subsequent IAA were assessed using Kaplan-Meier plots and compared using the log-rank test. All statistical analyses were conducted using the SAS software package, version 9.1 (2002, SAS Statistical Institute, Cary, North Carolina).

## 3. Results

### 3.1. Baseline Characteristics of the Study Patients

Successful implantation of catheters was performed in 202 patients who received CAPD during the 2-year minimum follow-up period in our institution. Forty (40/202, 19.8%) of the 202 patients had a history of abdominal surgeries. The indications for surgery in these 40 patients included appendectomy (11/40), caesarean section (11/40), cholecystectomy (4/40), transabdominal nephrectomy (4/40), transabdominal procedures of the ureter or bladder (3/40), abdominal hysterectomy (1/40), and/or salpingectomy (1/40), and oophorectomy (1/40), as well as procedures on the diaphragm (1/40), stomach (1/40), spleen (1/40), or intestines (1/40).

The results of the comparative analysis between the 38 patients with subsequent IAA and the remaining 186 patients without IAA are shown in Table 1. Mean patient age was 50.7±15.3 (range 8-83) years old and 51.2% were female. Except gender and age, there were no significant differences between the patients with or without IAA, in terms of overall 1- and 2-year catheter survival times, postoperative follow up time, initial PD prescription, and adequate indices at presentation (i.e., dialysate infusion volume, renal and peritoneal Kt/V urea, and WCcr), nPCR, and nutrition status (Tables 1 and 2).

### 3.2. Changes of PD Adequacy Indices in Patients with and without Subsequent Intra-Abdominal Adhesions (IAA) at Baseline and Two-Year Follow-Up Period

In order to elucidate whether the long-term PD adequacy indices differed between the two patient groups, we further analyzed PD adequacy indices after the 2-year minimum follow-up period (Table 2). The results demonstrated that 24-hour dialysate volume infusions were significantly lower in IAA patients compared to those subjects without adhesions. Furthermore, peritoneal WCcr was also significantly lower in IAA patients. Other parameters including renal, peritoneal Kt/V urea, nPCR, and residual renal WCcr showed no significant differences between the two patient groups.

In order to exclude the possible effects of prior surgical procedures on PD adequacy indices, the hypothesis that the level of PD adequacy indices were equal between prior surgery procedures was tested using ANCOVA. Univariate analysis of covariance between the two treated groups at the two different time points (baseline and 2-year follow-up period) showed that none of the PD adequacy indices were statistically different between the two groups.

### 3.3. The Relationship between Subsequent Intra-Abdominal Adhesions and Prior Surgical Procedures

In order to determine the relationships between previous abdominal surgery, IAA, adhesions, and clinical outcomes, our patients were divided into four observation groups (previous abdominal surgery with IAA, previous abdominal surgery without IAA, no previous abdominal surgery with IAA, and no previous abdominal surgery without IAA) (Table 3). Among patients with previous abdominal surgery, 16 (40%) and 24 (60%) patients had and did not have abdominal adhesions, respectively. However, 22 (13.6%) of the 162 patients without a history of abdominal surgery had abdominal adhesions (P<0.0001, chi-Square test). The differences in operation times for catheter implantation among the four groups were not statistically significant (P=0.670, one-way ANOVA). Furthermore, the differences in ages among the four groups (previous abdominal surgery with IAA, previous abdominal surgery without IAA, no previous abdominal surgery with IAA, and no previous abdominal surgery without IAA) were statistically significant (P=0.023, one-way ANOVA). The occurrence of PD peritonitis among the four groups was not statistical significant (P=0.161, chi-Square test).

### 3.4. The Relationship between Subsequent Intra-Abdominal Adhesions and Outcomes

**Figure 1:**
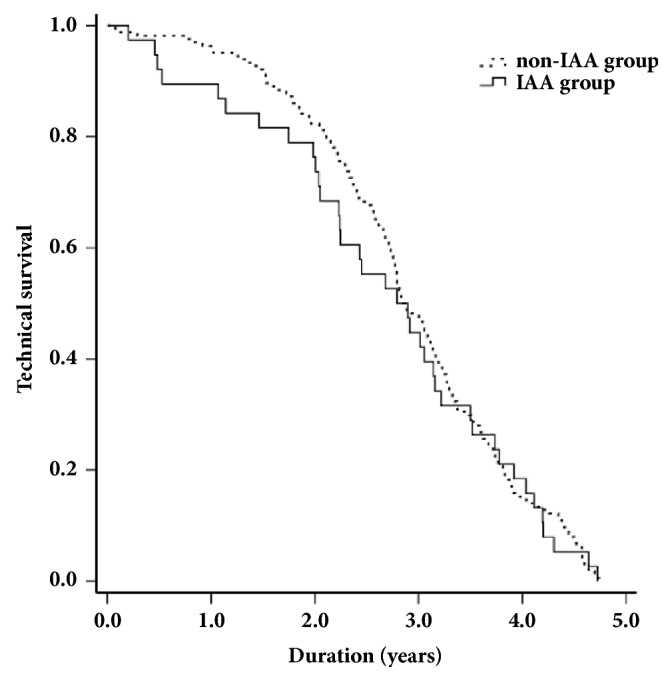
Kaplan-Meier Plots indicating the percentage of technical survival in the 202 patients with maintenance peritoneal dialysis. The patients were divided into those with and without subsequent intra-abdominal adhesion. The P value was obtained by log-rank comparison of data.

For patients with or without intra-abdominal adhesions, we calculated the Kaplan-Meier estimates for survival at the different times, which did not demonstrate statistical significance (P=0.716, log-rank test) (Figure 1).

## 4. Discussion

As a therapeutic modality, continuous ambulatory or automated PD is an increasingly popular option with widespread applications for patients with advanced renal failure. Exclusive PD advantages include less expense, simplicity, better preservation of renal function, and better quality of life [23–25]. However, IAA represents a major unsolved problem in adequate PD performance. Several challenges need to be addressed in order to prevent adhesions, including complicated catheter placements due to adhesive scarring, malpositioning, migration, obstruction or kinking of the tubing, limited intraperitoneal space for adequate dialyzable space, and inadequate flow function resulting from blocked drainage holes [25].

The roles of previous abdominal surgery on the presence of IAA remain controversial. Some physicians believe that the adhesions resulted from previous abdominal surgeries, which lead to a series of complications, such as the increased probability of peritonitis, prolonged hospitalizations, inadequate clearance due to insufficient infusion volume and mechanical obstruction [11–17].

The present study examined the effect of the presence of intra-abdominal adhesion or on both dialysis adequacy and outcomes in incident peritoneal dialysis patients and has three major findings. First, PD adequacy indices including both 24-hour dialysate volume and peritoneal WCcr L/week/ 1.73m^2^ were significantly low in patients with compared to those without subsequent IAA. Second, patients who had previous abdominal surgeries had higher risks of subsequent IAA, especially those with higher mean ages. Third, although patients who had subsequent IAA had lower PD adequacy, technical failures in PD maintenance did not result in significant differences at the 2-year minimum follow-up study.

Our study has several limitations. First, this is a retrospective analysis and is therefore subject to bias of unmeasured factors. The surgical procedures for the previous abdominal surgeries (e.g., location, type of lesions, and surgical procedures) were different for each patient. Second, there is a variety of most adequacy parameters. Besides previous abdominal surgery, several mechanisms are implicated in the development of IAA, including the occult infectious process, the alteration of peritoneal permeability, and exposure of biological damage resulting from the glucose-base dialysate [18, 26]. The commencement of surgical procedures for catheter insertion in those patients with advanced renal failure who require PD differed for each patient according to the preference of his/her doctor. Third, the reasons for technical survival are multifactorial (e.g., presence of mechanical and infectious complications, patient's conditions and self-care ability, and preference of patients for the choice of dialysis). According to International Society of peritoneal dialysis (ISPD) treatment guideline, we maintain the peritoneal dialysis in the low dialysis adequacy of the IAA group. Finally, the short follow-up period and the assessment of adequacy of PD therapy relied on a longer follow-up period.

In conclusion, subsequent IAA is not rare, especially in high-risk patients, including those with previous abdominal surgeries and higher mean ages. Although lower PD adequacy after IAA was found, technical failures did not show significant differences at the 2-year minimum follow-up study.

## Figures and Tables

**Table 1:** Comparisons of the baseline characteristics of PD patients with and without subsequent IAA after catheter insertion at presentation.

	*With subsequent IAA* ^*a*^	* Without subsequent IAA* ^*a *^	*Total *	*p-value * ^Ϯ^
	*(mean ± SD) *	*(mean ± SD)*		
*Patients [n (%)] *	*38 (18.8%) *	*164 (81.2%) *	*202*	
*Gender (female) *	*28 (84.89%) *	*81 (54.36%) *	*109 (51.2%) *	*0.007*
*Age (years) *	*55.4±15.5*	*49.3±14.0*	*50.7±15.3*	*0.018*
*Postoperative follow up (years) *	*2.9±1.0*	*2.7±1.2 *	*2.8±1.2*	*0.364*
*Overall 1- & 2-year catheter survival *	*90% & 79% *	*96% & 86% *	*95% & 85% *	*0.173*
*Peritonitis [n (%)] *	*19 (50.0%) *	*55 (27.2%) *	*74 (36.6%) *	*0.058*
*Admission [n (%)] *	*21 (55.3%) *	*83 (50.6%) *	*104 (51.5%) *	*0.062*

*IAA: intra-abdominal adhesions.*

*Ϯ  =  Baseline characteristics between two patient groups at presentation were compared by way of independent t-test.*

IAA^a^  = Intra-abdominal adhesions.

**Table 2:** Comparisons of the PD adequacy indices in patients with and without subsequent IAA at presentation and the 2-year minimum follow-up period.

	*With subsequent IAA* ^*a*^		*Without subsequent IAA* ^*a*^		*p-value* ^*b*^
	*(mean ± SD)*		*(mean ± SD)*		
	*At presentation*	*Follow-up*	*At presentation*	*Follow-up*	
*Body weight (kg)*	*55.64±13.33*	*56.82±12.98*	*58.10±11.42*	*60.34±12.27*	*0.815*
*Body height (cm)*	*156.73±9.35*	*156.35±9.45*	*158.50±16.97*	*158.38±16.96*	*0.637*
*24-hour urine volume*	*1.09±0.84*	*0.73±0.88*	*1.04±0.65*	*0.61±0.56*	*0.779*
*24-hour dialysate volume*	*6.53±1.63*	*7.69±2.35*	*7.17±1.94*	*9.15±2.54*	*0.003*
*Peritoneal Kt/V urea *	*1.48±0.39*	*1.57±0.40*	*1.45±0.32*	*1.72±0.39*	*0.051*
*Residual renal Kt/V urea *	*0.83±0.65*	*0.44±0.50*	*0.67±0.49*	*0.35±0.32*	*0.092*
*Total Kt/V urea *	*2.30±0.56*	*2.00±0.34*	*2.12±0.48*	*2.04±0.39*	*0.095*
*Peritoneal WCcr L/week/ 1.73m* ^*2*^	*33.81±9.43*	*36.69±10.57*	*35.59±9.02*	*42.28±12.50*	*0.018*
*Residual renal WCcr L/week/1.73m* ^*2*^	*37.78±29.77*	*19.99±21.97*	*32.48±24.60*	*16.69±15.34*	*0.405*
*Total WCcr L/week/1.73m2 *	*80.44±26.82*	*62.66±21.02*	*73.84±23.41*	*61.40±17.03*	*0.375*
*nPCR *	*1.12±0.39*	*1.02±0.28*	*1.09±0.27*	*1.01±0.25*	*0.937*

IAA^*a*^*: *Intra-abdominal adhesions.

b = PD adequacy indices at two time period (at presentation and 2-year follow-up) between the two patient groups by means of repeated measures of ANOVA.

**Table 3:** Comparison between subsequent intra-abdominal adhesion and prior surgical procedures.

	*Surgery* ^a^ *(n=40)*		*Non-surgery (n=162)*		*p-value*
	*With adhesion *	*Without adhesion*	*With adhesion *	*Without adhesion*	
*Patients numbers (n)* ^*d*^	*16 (40 %)*	*24 (60 %)*	*22 (13.6 %)*	*140 (86.4 %)*	*<0.001*
*Age (years)* ^b^	*60.7±12.8*	*50.5±12.7*	*51.6±16.4*	*49.1±14.2*	*0.023*
*Operation time (minutes)* ^c^	*81.4±43.4*	*71.3±34.2*	*76.7±24.5*	*72.4±30.2*	*0.670*
*PD peritonitis [n (%)]* ^*d*^	*8 (21.1%) *	*8 (4.9%) *	*11 (28.9%) *	*47 (28.7%) *	*0.161*

a = previous abdominal surgery.

b and c = the age and operation time among four groups were compared by means of one-way ANOVA.

d =the differences among four groups were compared by means of a Chi-Square test.

## Data Availability

The data used to support the findings of this study are available from the corresponding author upon request.

## Abbreviations

PD: Peritoneal dialysis
IAA: Intra-abdominal adhesions
ESRD: End stage renal disease
WCcr: Weekly creatinine clearance
BMI: Body mass index
nPCR: Normalized protein catabolic rate
ANCOVA: Analysis of covariance.
